# COVID-19-associated schizophrenia-like psychosis during the COVID-19 pandemic

**DOI:** 10.1192/j.eurpsy.2023.1689

**Published:** 2023-07-19

**Authors:** L. Bravve, N. Zakharova

**Affiliations:** Psychiatric Hospital no. 1 Named after N.A. Alexeev of the Department of Health of Moscow, Moscow, Russian Federation

## Abstract

**Introduction:**

The viral theory of the development of psychoses was formulated in the 19th century, but the role of viral agents in the pathogenesis of psychoses remains a matter of debate. In the context of the 2020 pandemic, the results of several papers on the characteristics of psychotic disorders in COVID-19 have been published. Coronaviruses are neuroinvasive, capable of affecting mental and body functions.

**Objectives:**

The aim of the study was to identify patients with acute psychosis without a history of mental disorders in order to test the hypothesis about the possibility of manifestation of schizophreniform psychosis due to a viral attack.

**Methods:**

Within a year and a half, 50 patients with mental disorders associated with COVID-19 were identified. The reason for hospitalization in a psychiatric hospital is the development of acute schizophreniform psychosis against the background of a new coronavirus infection. The diagnosis was verified according to traditional clinical criteria and modern psychometric tools. Inclusion criteria: no previous visits to a psychiatrist; verified acute polymorphic psychotic disorder and coronavirus infection COVID-19 (ICD-10); manifestation of psychosis against the background of infection with COVID-19; age 18-60 years; consent to participate in the study. Exclusion criteria: signs of organic brain damage; indications of substance abuse; delirium of any etiology; somatic pathology in the stage of decompensation.

**Results:**

27 women (54%) and 23 men (46%), aged 20 to 57 years (average age 34.5±7.6), of which 18 people (36%) worked, 2 (4%) studied , 29 (58%) people are unemployed. 27 people (54%) are single, 16 (32%) are married, 7 (14%) are divorced; 18 people (36%) were raising children, which indirectly indicated a relatively favorable premorbid functioning. At the time of the examination, the condition was determined by polymorphic psychotic symptoms with a predominance of dissociative-delusional, paraphrenic and oneiric phenomena with plots containing plots of infection and the spread of coronavirus. At the same time, the sum of PANSS is from 53 to 130 (85) points, including PANSS P - from 15 to 37 (27), PANSS N - from 7 to 44 (18), PANSS G - from 23 to 57 (39.9 ); P1 - 5.7; P3 - 5.1. When comparing the obtained data with the world experience, we found some differences. Delusional symptoms were diagnosed twice as rarely, cases with tactile hallucinations were not detected, but catatonic symptoms were observed twice as often, and manic arousal was twice as rare. In general, the studied data are comparable with the results published in the world practice.

**Conclusions:**

The unique experience of the coronavirus pandemic will allow us to assess the influence of environmental factors, namely the role of infections, in the manifestation or predisposition to schizophrenia spectrum disorders. It is planned to evaluate the dynamics of the course of the disorder after the relief of the acute period.

**Disclosure of Interest:**

None Declared

